# Thermo-Mechanical Fluid–Structure Interaction Numerical Modelling and Experimental Validation of MEMS Electrothermal Actuators for Aqueous Biomedical Applications

**DOI:** 10.3390/mi14061264

**Published:** 2023-06-17

**Authors:** Thomas Sciberras, Marija Demicoli, Ivan Grech, Bertram Mallia, Pierluigi Mollicone, Nicholas Sammut

**Affiliations:** 1 Department of Mechanical Engineering, Faculty of Engineering, University of Malta, MSD 2080 Msida, Malta; pierluigi.mollicone@um.edu.mt; 2 Institute for Sustainable Energy, University of Malta, MXK 1531 Marsaxlokk, Malta; marija.demicoli@um.edu.mt; 3 Department of Microelectronics and Nanoelectronics, Faculty of Information and Communications Technology, University of Malta, MSD 2080 Msida, Malta; ivan.grech@um.edu.mt (I.G.); nicholas.sammut@um.edu.mt (N.S.); 4 Department of Metallurgy and Materials Engineering, Faculty of Engineering, University of Malta, MSD 2080 Msida, Malta; bertram.mallia@um.edu.mt

**Keywords:** numerical modelling, electrothermal actuator (ETA), V-shaped driver, microelectromechanical system (MEMS), fluid–structure interaction, finite element, finite volume, submerged, underwater, aqueous

## Abstract

Recent developments in MEMS technologies have made such devices attractive for use in applications that involve precision engineering and scalability. In the biomedical industry, MEMS devices have gained popularity in recent years for use as single-cell manipulation and characterisation tools. A niche application is the mechanical characterisation of single human red blood cells, which may exhibit certain pathological conditions that impart biomarkers of quantifiable magnitude that are potentially detectable via MEMS devices. Such applications come with stringent thermal and structural specifications wherein the potential device candidates must be able to function with no exceptions. This work presents a state-of-the-art numerical modelling methodology that is capable of accurately predicting MEMS device performance in various media, including aqueous ones. The method is strongly coupled in nature, whereby thermal as well as structural degrees of freedom are transferred to and from finite element and finite volume solvers at every iteration. This method therefore provides MEMS design engineers with a reliable tool that can be used in design and development stages and helps to avoid total reliability on experimental testing. The proposed numerical model is validated via a series of physical experiments. Four MEMS electrothermal actuators with cascaded V-shaped drivers are presented. With the use of the newly proposed numerical model as well as the experimental testing, the MEMS devices’ suitability for biomedical applications is confirmed.

## 1. Introduction

### 1.1. MEMS for Biomedical Applications and Device Specifications

The ability to manufacture devices with micron-sized features and footprints has placed MEMS at the forefront of innovation in the biomedical industry, where such devices are exploited for procedures such as drug delivery [1,2], micromanipulation [3,4,5] and the mechanical characterisation of biological cells [4,6,7,8].

Because MEMS devices are heavily investigated in biomedical engineering-oriented research, there is a large variety of MEMS structures/mechanisms and grasping methodologies, most of which share a common goal—micromanipulation for cell characterisation [9,10]. Previous work has deduced that there are certain pathological conditions which inherently alter a cell’s rheology; that is, the cell’s characteristics (such as mechanical stiffness), under the influence of such a disease, drift from that of a healthy one [11]. The presence of such a characteristic change is often referred to as a ‘biomarker’ for concerned analysts [12]. One such condition is sickle cell anaemia (SCA), whereby under the influence of this pathological condition, a human red blood cell (RBC) exhibits an increase in stiffness in a quantifiable magnitude when compared with a healthy red blood cell [6,13]. Although there are a number of already accepted as well as emerging technologies to physically test for such conditions such as ektacytometry [14,15], atom force microscopy (AFM) [16], and optical tweezers [17,18], MEMS actuators pose themselves as potentially cheaper and more promising candidates for the task.

MEMS actuators may be designed and tailored in such a manner so as to impart a mechanical strain on a test subject (in this case, a human RBC), and the diagnosis may be performed either via postprocessing routines [18,19] or inbuilt sensing [20]. The preservation of an RBC’s original integrity during the test procedure is of utmost importance, and this in turn imparts a set of principle design specifications on the MEMS devices. These criteria are outlined below, and the outcome of the criteria shall be a set of specifications by which the MEMS must abide in order to qualify as a prospective human RBC diagnostic tool.

#### 1.1.1. Dimensional Characteristics

The major diameter of a human RBC is typically in the order of 10 µm, and capillary cross-sectional diameters may be as small as 3 µm [21]. This implies that at minimum, the MEMS device must be able to compress a cell by 7 µm across its major diameter. The standard configurations in which MEMS actuators are typically designed are twofold: they are either normally closed or normally open. As the name implies, a normally closed actuator is one that is closed at rest and is forced to open when subjected to a stimulus. Typically, the rest opening length would be in the order of 3 µm, and the opening distance when stimulated would be marginally above 10 µm. Such a design typically requires a structure that is stiff enough to impart the required deformations onto the test subject as it returns to its rest position. The opposite holds for a normally open actuator, where the gripping area is opened when no stimulus is present and is forced to close when activated. This work shall focus on normally open MEMS electrothermal actuators (ETAs), wherein the input power/stimulus shall be electrical in nature.

#### 1.1.2. Temperature

A human RBC has a defined range of temperatures within which it is viable for testing purposes (temperature range between 22 °C and 40 °C) [22,23,24]. The upper temperature limit implies that not only must the testing environment be maintained below this temperature, but also that the device must not generate a temperature that is higher than the prescribed limit at the test location during operation, as this may damage the cell and therefore give rise to erroneous readings.

#### 1.1.3. Test Medium

When RBCs are removed from plasma and placed in a testing medium, the medium tends to determine the shape of the RBC [25]. Therefore, another requirement for maintaining a cell’s original integrity is the need for it to be kept immersed in what is termed an isotonic solution [26]. In the case of a human RBC, 0.9% NaCl is considered an isotonic solution [27]. Because the MEMS device architecture occupies micron-sized footprints, it is virtually impossible to submerge merely a portion of the device, which gives rise to perhaps the most challenging specification of MEMS, i.e., that they need to function when completely submersed within the cell that contains fluid. Given the large thermal conductivity and convection coefficients that are associated with water-based solutions, a previous work deduced that electrothermal MEMS actuators function at a capacity of 6% (per applied volt) of what they would in the air when they are operated in water [28].

Furthermore, besides the already mentioned criterion that the test environment and location must not exceed a temperature of 40 °C, no location across the entirety of the device must reach or exceed temperatures of 100 °C so as to avoid boiling of the water, as gas evolution in close proximity to the test site may hinder measurements.

It has been documented that there are other parameters that affect the mechanical stiffness of RBCs; however, they are somewhat outside the scope of this work. Such parameters include RBC storage temperature as well as duration [29,30].

The operation of MEMS ETAs in aqueous electrolytes has other challenges that are associated with it; among them are electrochemical corrosion and electrolysis of the solution [31,32]. Corrosion of MEMS devices has posed itself as a critical reliability concern, especially when large voltages are applied on close proximity electrodes [33]. Although corrosion is of greater concern for electrostatic actuators in aqueous media, mainly due to their tightly spaced comb drives and high potentials, the corrosion of electrothermal MEMS devices may also be an issue [31]. Electrolysis of the fluid must also be avoided so as to not hinder the cell manipulation or characterisation procedure via gas evolution. Relying on a DC source for the underwater operation of MEMS devices limits users and designers to a maximum operating voltage of 1.23 V, at which point the initiation of the electrolysis of DI water becomes a concern [31,34]. Mukundan et al. [32] propose avoiding such electrochemical phenomena by supplying the MEMS devices with high-frequency (100 kHz) AC pulses having a mean voltage of 0 V as opposed to opting for a DC source. MEMS ETA response will therefore be proportional to the RMS of the input power; hence, in this way, users may supply devices with higher powers to achieve the required outputs.

Due to the fact that device performance prediction tools specifically designed for biomedical applications are still relatively in their infancy at present, this work aims at presenting a numerical modelling technique that is capable of accurately predicting the electro-thermo-mechanical performance of MEMS ETAs for underwater biomedical operations. Devices that satisfy all of the above-mentioned criteria are also presented.

## 2. Fabrication Process Overview

**Figure 1:**
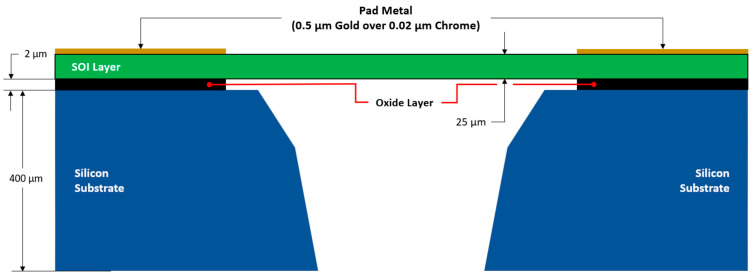
Typical SOIMUMPs structure in a section view. Note: image is not to scale. Figure is based on the procedures described in [35].

The micromachining process selected in this work is the commercially available silicon-on-insulator multi-user MEMS processes (SOIMUMPs™) [35]. The reason for opting for this process, other than the fact that it is commercially available, is that it offers structurally robust designs due to the significant thickness of the SOI layer. This thickness, together with the material properties of the SOI, creates excellent out-of-plane stiffness and hence makes this process ideal for suspended type structures/actuators with large floating masses. Figure 1 shows a section view of a typical SOIMUMPs structure. With reference to Figure 1, the process stack is composed of a 25 µm thick n-type semiconductor, which is the SOI layer. It is in this layer that the desired electrical circuitry as well as the structural members of the devices are designed. Below the SOI layer is a 1 µm thick silicon oxide layer which electrically isolates the SOI layer from the substrate. The substrate is a 400 µm thick silicon base, which has a much higher electrical resistance compared with the SOI. To apply an external electrical power source to the circuit, the addition of a pad metal layer on top of the SOI layer is possible. This layer is composed of a 0.02 µm chromium under-strike and is coated with 0.5 µm of gold.

**Figure 2:**
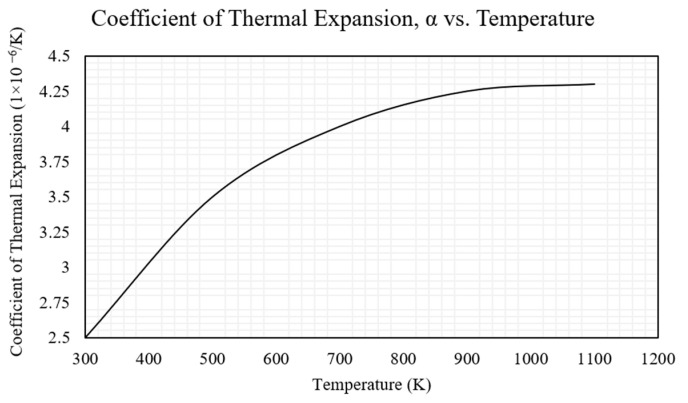
SOI coefficient of the thermal expansion vs. the temperature graph as derived from [35] and extracted from [8]. © 2023 IEEE. Reprinted with permission from T. Sciberras, P. Mollicone, M. Demicoli, I. Grech, N. Sammut, and B. Mallia, “Experimental and Numerical Analysis of MEMS Electrothermal Actuators with Cascaded V-shaped Mechanisms,” *2022 Symposium on Design*, *Test*, *Integration and Packaging of MEMS/MOEMS (DTIP)*, Pont-a-Mousson, France, 2022, pp. 1–5 [8].

Refer to Table 1 and Figure 2 for the material properties of both the SOI layer as well as the pad metal layer; these properties were used in the numerical models described later. Note that when simulating the silicon substrate, an electrical resistivity of 50,000 Ω·µm was used [35]. Moreover, with reference to Table 1, a low resistivity SOI layer was used as this layer was used for the thermal expansion (via Joule heating) and hence the tip displacement. This low resistance compared with that of the test medium (water) is also required so that the electrical current preferentially passes through the semiconductor instead of the fluid.

## 3. Electrothermal MEMS Actuators’ Actuating Principles and Device Designs

### 3.1. MEMS Electrothermal Actuators’ Working Principles

The abundance of MEMS ETA structures, configurations, and functional characteristics is particularly apparent nowadays [36]. The most commonly researched configurations include the V-shaped ETA [37,38], hot-and-cold arm ETA [11,39], and cascaded V-shaped ETA [40,41]. The latter driver is the primary driver used in the devices discussed here.

**Figure 3:**
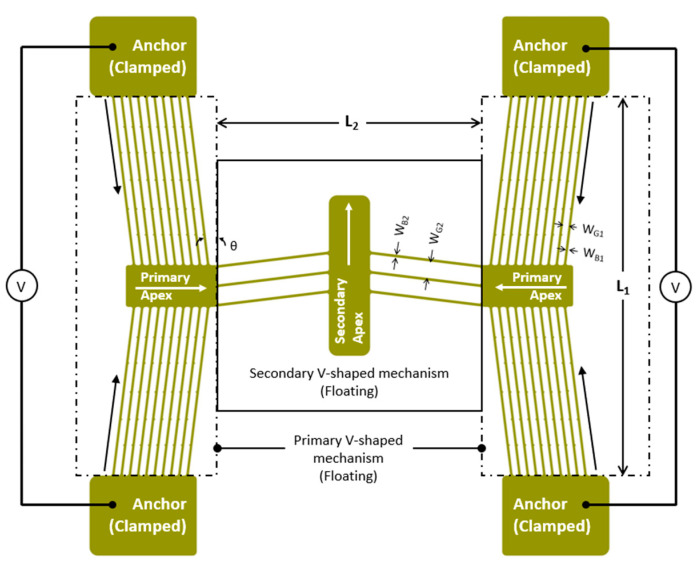
Graphical representation of a cascaded V-shaped driver in the plan view. Single arrow lines indicate the direction of motion once an external power source is applied to the primary drivers. Figure as adapted from [8]. © 2023 IEEE. Reprinted with permission from T. Sciberras, P. Mollicone, M. Demicoli, I. Grech, N. Sammut, and B. Mallia, “Experimental and Numerical Analysis of MEMS Electrothermal Actuators with Cascaded V-shaped Mechanisms,” *2022 Symposium on Design*, *Test*, *Integration and Packaging of MEMS/MOEMS (DTIP)*, Pont-a-Mousson, France, 2022, pp. 1–5 [8].

With reference to Figure 3, the cascaded V-shaped driver is broadly composed of two opposing, active V-shaped drivers that are interconnected by a single passive V-shaped mechanism placed normal to the active ones [40,41,42]. A single, stand-alone V-shaped mechanism is devised by connecting two mechanically clamped anchor regions via a series of floating beams and a central shuttle [8,37]. External energy is supplied to the driver by subjecting it to a potential difference across the anchors. Once the potential difference is present, current flows through the SOI and Joule heating is generated. In turn, the semiconductor experiences an increase in temperature, which instigates thermal expansion and therefore motion. In a classical, symmetric V-shaped ETA, the maximum temperature develops at the centre of the shuttle. This location is also the region of largest displacement, the direction of which is parallel to the mentioned shuttle [8,31,37,43]. Conversely to the hot-and-cold arm ETA, a V-shaped ETA actuates via global Joule heating rather than via differential heating, thus making the V-shaped driver better suited for applications that involve the driver having to function while submerged in fluids of higher thermal conductivity, such as water [31]. Although design optimisation loops are common during the design stages of MEMS ETAs, a stand-alone V-shaped mechanism tends to generate relatively low displacements at the central shuttle’s apex, and depending on the application, secondary amplification mechanisms are often required [8].

The cascaded V-shaped driver offers an initial amplification method through the use of the passive secondary V-shaped structure. Like the active/primary mechanisms, the secondary mechanism is also floating. It is geometrically perpendicular to the primary drivers and is strategically placed at the dead centre of the global mechanism. The mechanism is activated by subjecting the two primary drivers to a potential difference, typically of equal magnitude. Although typically combined to stand-alone V-shaped mechanisms, other lever type and flex features are possible and can be incorporated to a cascaded V-shaped driver to serve as additional mechanical amplifiers [8,36,44]. As evident in the subsequent sections, other than merely serving for mechanical gain purposes, this secondary mechanism also helps in reducing the steady-state temperature at the test location due to a longer thermal path from the primary apexes.

### 3.2. Device Designs and Configurations

**Figure 4:**
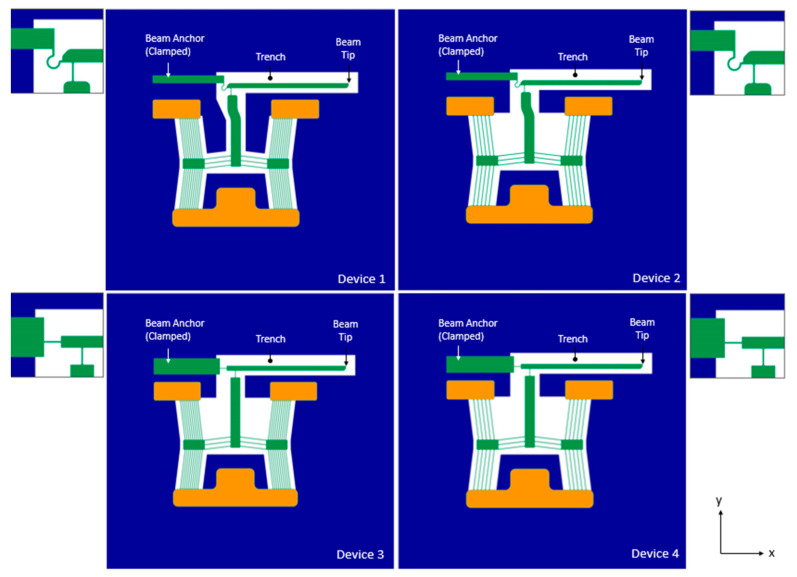
Geometrical models of device configurations (in plan view) analysed in this work. Image as adapted from [8]. Note that geometry in blue represents the substrate material, green represents the SOI, and orange represents the pad metal. Outset images are detailed views of the hinge layout of the individual devices. © 2023 IEEE. Reprinted with permission from T. Sciberras, P. Mollicone, M. Demicoli, I. Grech, N. Sammut, and B. Mallia, “Experimental and Numerical Analysis of MEMS Electrothermal Actuators with Cascaded V-shaped Mechanisms,” *2022 Symposium on Design*, *Test*, *Integration and Packaging of MEMS/MOEMS (DTIP)*, Pont-a-Mousson, France, 2022, pp. 1–5 [8].

This work shall focus on the device configurations and characteristics, which were presented in [8]. All designations and variables, including the material properties and structural configurations, are carried over into this text. Refer to Figure 4 for a graphical representation of all four devices. To recap, devices 1 and 3 make use of ten primary beams per driver but have different hinge layouts connecting the amplification beam to the secondary apex and beam anchor. Similarly, devices 2 and 4 also have the same number of beams at five per primary driver, and they have differing hinge layouts. Moreover, devices 1 and 2 have the same hinge layout, which differs from that of devices 3 and 4 [8]. Table 2 summarises all geometric variables associated with each device.

With reference to Figure 4, when the devices are subjected to a potential difference across each of their primary drivers, Joule heating forces the left and right primary apexes to displace in the positive and negative *x*-axis, respectively. The displacement of primary apexes in turn forces the secondary apex to displace upwards. In all device cases, an amplification beam is connected to the secondary apex by a slender strut. The beam is also clamped to the substrate by a hinge feature. When the secondary apex displaces in the positive *y*-axis, this causes the beam to rotate about the hinge and achieve a positive tip displacement that is also in the *y*-axis.

## 4. Numerical Modelling

### 4.1. General

The strict specifications imparted on devices, especially for those intended for biomedical applications as described above, give rise to the need for accurate and reliable performance prediction. Numerical models help assist MEMS design engineers devise complex micro-mechanisms to reliably predict their device’s function in numerous applications and environmental conditions and not rely entirely on physical testing. Very often, electrothermally activated MEMS design engineers resort to virtually characterising devices using finite element solvers, wherein heat lost by the device via convection is either completely omitted or applied simply as a constant boundary condition [45,46,47]. Other methods assume that convection can be replaced by conduction to the substrate [48,49,50]. While these assumptions may suffice in simpler scenarios such as operation in still air, their suitability is questionable for more complex functions where the device may be submerged in highly conductive, viscous fluids and/or where the device may be exposed to fluid flow where the significance of FSI is quantifiable. For this reason, the use of finite volume solvers in strongly coupled analyses of microdevices has recently gained interest [37,51]. Despite their large computational expense, they provide designers with higher-fidelity analyses for the more complex environmental conditions. Liu et al. [51] made use of a thermo-mechanical coupling to develop a damping model for an electrothermal MEMS micromirror operating in liquid paraffin; little information, however, is given regarding the numerical modelling procedure itself. Additionally, the electrothermal multiphysics coupling in the finite element domain was not considered, but rather the actuating element was clamped at a constant temperature, which is not the case in the method being proposed here. This electrothermal consideration in the finite element domain is an important element to consider as it sheds light on the device’s required power consumption to perform the intended task.

This section is dedicated to describing the state-of-the-art numerical modelling methodology that is being proposed in this work. This method shares many similarities with the two-way system coupling method presented in [37], where thermal coupling between a finite element and finite volume solvers was invoked. Here, however, thermo-mechanical coupling is utilised, meaning that the mechanical fluid–structure interaction (FSI) is calculated together with the thermal one. This makes the newly proposed method ideal for simulating MEMS device performance in fluids of higher viscosity where the mechanical resistance that is imparted onto the device by the fluid is no longer negligible. The method described herein was implemented using Ansys^®^ Academic Research Mechanical, Release 21.1.

### 4.2. Thermo-Mechanical Fluid–Structure Interaction Numerical Modelling

**Figure 5:**
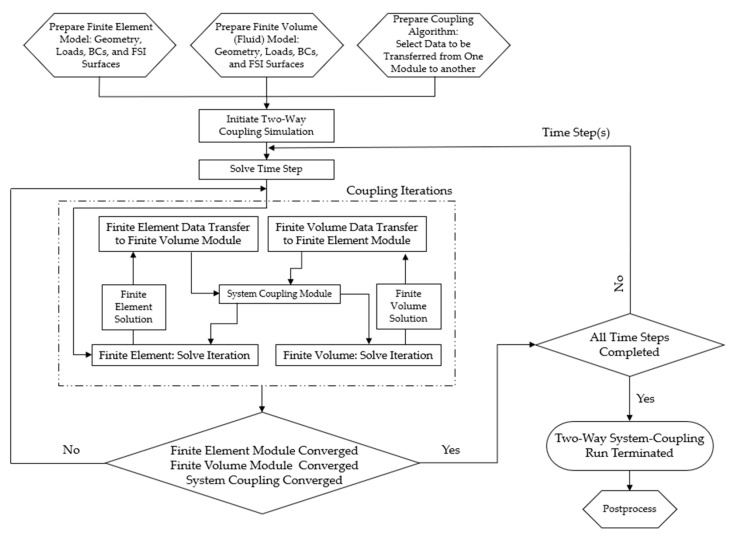
Thermo-mechanical FSI—Process flow and setup. Process flowchart depicted as a development of work from that presented in [37].

Similarly to the two-way coupled analysis presented in [37], the method proposed in this work includes two types of numerical models: one solved in a finite element domain and the other in a finite volume domain. The finite element domain is the one where the device geometry is contained and discretised into finite elements having a set of degrees of freedom that depend on the required physics. The finite volume domain is where the surrounding fluid is defined and discretised. The pair are linked via the ‘system coupling’ algorithm provided by the software, and the solutions of both are calculated concurrently per iteration. One major difference the proposed method has when compared with that of [37] is that the datasets being transferred during iterations are broader, as more variables are linked and calculated at the fluid–structure interface. The process flow and sequence of events for setting up such an analysis is summarised in Figure 5.

#### 4.2.1. Finite Element Module—Model Setup

With reference to Figure 5, the device models were set up as transient structural analyses. The geometries consisted of the substrate material in proximity to the devices, the SOI layer, and the gold layer only. As described in [37], the chromium under-strike of the pad metal was not included in the numerical models because its thickness is negligible compared with that of the gold; additionally, the electrical potential was assumed to not vary along the thickness of the pad metal. Moreover, the silicon oxide layer separating the SOI and the substrate was omitted from analysis for the same assumptions as discussed in [37] in that the substrate material was thermally clamped at a constant temperature for the full analysis duration. Although steady-state operation is of greatest interest in this work, a transient solver was used here because it offered more computational stability. Nevertheless, it is possible to investigate the steady-state response by looking at the time steps where results have stabilised with respect to time. In the work presented in [37], the device geometry was discretised with 20-node brick type elements having thermal and electrical degrees of freedom. Here, however, all component geometries (of all devices) were discretised into higher order, 10-node tetrahedral elements having displacement, temperature, and electrical degrees of freedom. The reason for opting for tetrahedral elements is mainly due to the more complex nature of the geometries, making discretisation more efficient.

**Figure 6:**
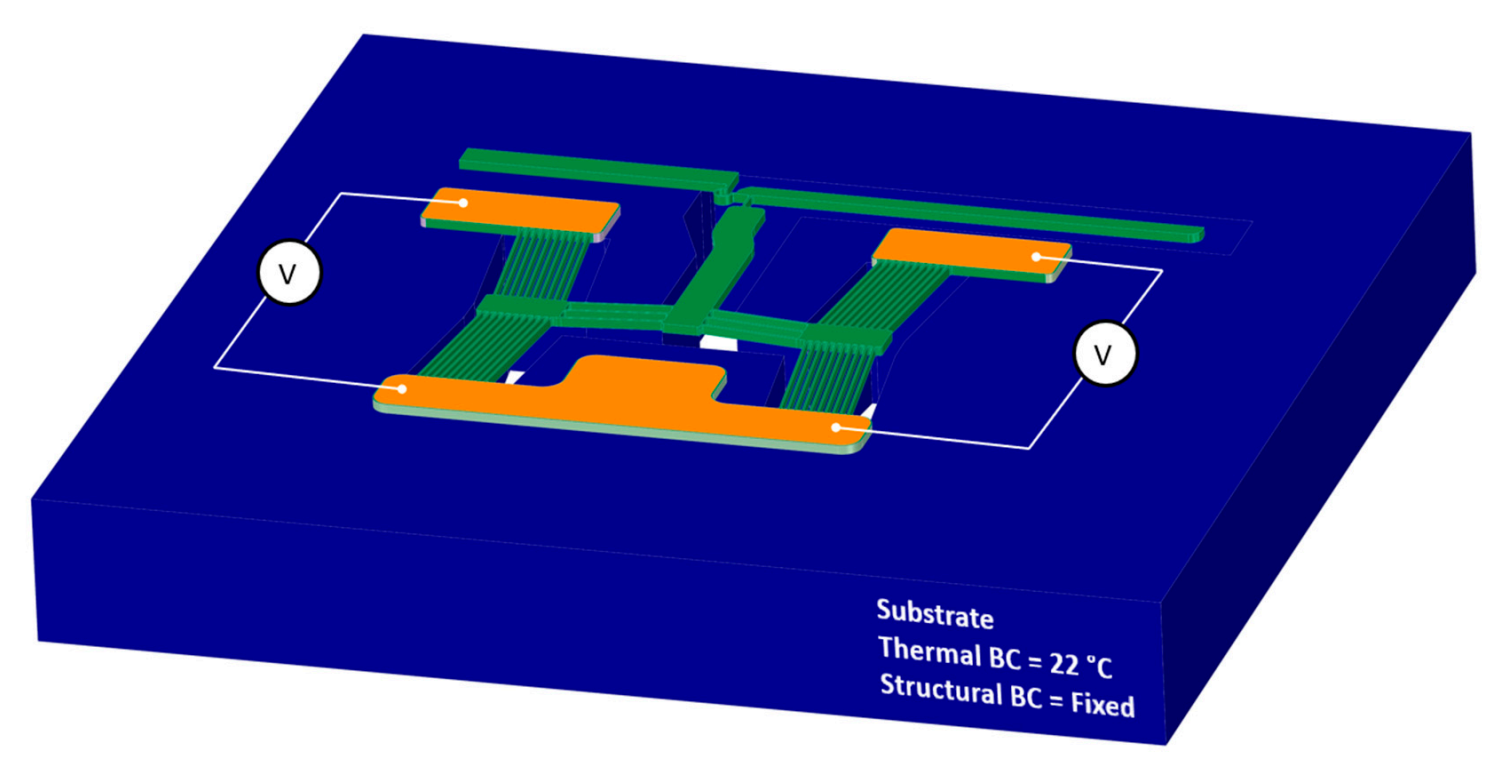
Computer-aided model of the finite element model setup. The device depicted is device 1; however, the representation is applicable to all devices. © 2023 IEEE. Reprinted with permission from T. Sciberras, P. Mollicone, M. Demicoli, I. Grech, N. Sammut, and B. Mallia, “Experimental and Numerical Analysis of MEMS Electrothermal Actuators with Cascaded V-shaped Mechanisms,” *2022 Symposium on Design*, *Test*, *Integration and Packaging of MEMS/MOEMS (DTIP)*, Pont-a-Mousson, France, 2022, pp. 1–5 [8].

With reference to Figure 6, the loads and boundary conditions applied in the finite element module of all devices are:

i. A thermal boundary condition applied on the entire substrate as thermally clamped at 22 °C for all time steps;
ii. The substrate was also mechanically clamped for all time steps, i.e., no translations are allowed;
iii. A potential difference (V) was applied across the primary apexes via the pad metal regions, as denoted in Figure 6. The voltage loads for all devices were swept from 0 V to 5 V in increments of 1 V.

The finite element solver calculates the electro-thermo-mechanical function of the device using Equation (1) [52].

(1) $$\left[\begin{array}{ccc} \left[M\right] & \left[0\right] & \left[0\right]\\ \left[0\right] & \left[0\right] & \left[0\right]\\ \left[0\right] & \left[0\right] & \left[0\right] \end{array}\right]\left\{\begin{array}{c} \left\{\ddot{u}\right\}\\ \ddot{\left\{T\right\}}\\ \left\{\ddot{V}\right\} \end{array}\right\}+\left[\begin{array}{ccc} \left[C\right] & \left[0\right] & \left[0\right]\\ \left[C^{\mathrm{tu}}\right] & \left[C^{t}\right] & \left[0\right]\\ \left[0\right] & \left[0\right] & \left[C^{v}\right] \end{array}\right]\left\{\begin{array}{c} \dot{\left\{u\right\}}\\ \dot{\left\{T\right\}}\\ \dot{\left\{V\right\}} \end{array}\right\}+\left[\begin{array}{ccc} \left[K\right] & \left[K^{\mathrm{ut}}\right] & \left[0\right]\\ \left[0\right] & \left[K^{t}\right] & \left[0\right]\\ \left[0\right] & \left[K^{\mathrm{vt}}\right] & \left[K^{v}\right] \end{array}\right]\left\{\begin{array}{c} \left\{u\right\}\\ \left\{T\right\}\\ \left\{V\right\} \end{array}\right\}=\left\{\begin{array}{c} \left\{F\right\}\\ \left\{Q\right\}\\ \left\{I\right\} \end{array}\right\}$$

where [M] is the structural mass matrix; $\{\ddot{u}$}, {$\ddot{T}$}, and {$\ddot{V}$} are the second-time derivatives of the displacement, thermal potential, and electrical potential vectors, respectively; [C] is the structural damping matrix; $\left[C^{t}\right]$ is the specific heat matrix; $\left[C^{\mathrm{tu}}\right]$ is the thermoelastic damping matrix; $\left[C^{v}\right]$ is dielectric permittivity coefficient matrix; $\dot{u}$, $\dot{T}$, and $\dot{V}$ are the first-time derivatives of the displacement, thermal potential, and electrical potential vectors, respectively; [K] is the structural stiffness matrix; $\left[K^{\mathrm{ut}}\right]$ is the thermoelastic stiffness matrix; $\left[K^{t}\right]$ is the thermal conductivity matrix; $\left[K^{\mathrm{vt}}\right]$ is the seebeck coefficient coupling matrix; $\left[K^{v}\right]$ is the electrical conductivity matrix; {u}, {T}, and {V} are the displacement, thermal potential, and electrical potential vectors, respectively; {F} is the load vector; {Q} is the heat flow vector; and {I} is the electrical current vector [52].

#### 4.2.2. Finite Volume Module—Model Setup

**Figure 7:**
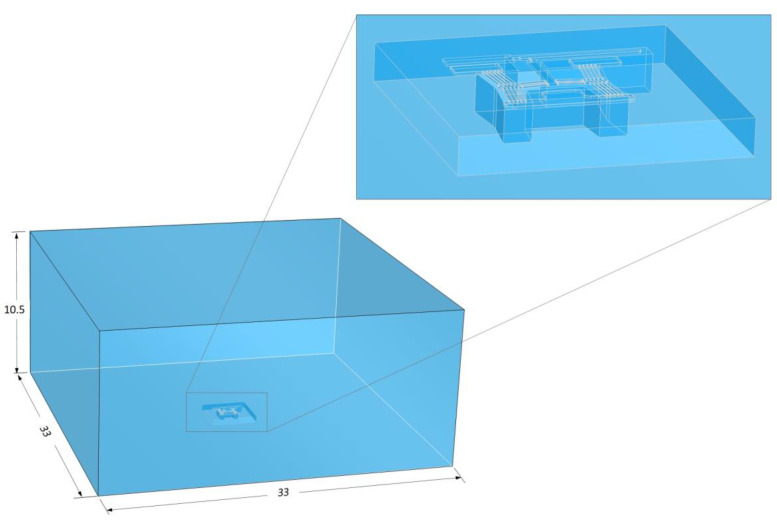
Computer-aided model of the finite volume domain as modelled for all device configurations. Bottom left: the full domain; top right: detailed view of the FSI region cut-out in the finite volume domain. Dimensions are in mm. The domain depicted is that of device 4.

**Figure 8:**
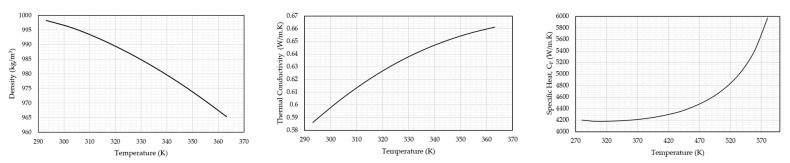
Temperature-varying physical properties of water as implemented in the finite volume numerical models. Density vs. temperature graph (**Left**), thermal conductivity vs. temperature graph (**middle**), and specific heat capacity at constant pressure (**right**) [54].

The finite volume models were implemented in Fluent^®^ software, release 2021 R1. The above-mentioned domain consisted of fluid around the MEMS devices and was modelled such that this domain was in excess of ten times the device’s major dimensions to allow for accurate heat transfer computations. Recall from [37] that the volume of the device and its components are not included in the fluid domain when executing such a coupled analysis. This holds for the proposed method as well, whereby the volumes of the finite element model are cut from the finite volume geometry. Refer to Figure 7 for a graphical representation of a typical finite volume domain. Because the surfaces of the cut-out region coincide with the outer surfaces of the device in the finite element domain, these shall be the locations of data transfer in the coupled analysis between the two modules. Because the finite element model has both thermal as well as structural degrees of freedom, the FSI surfaces at the cut-out within the finite volume domain were set to be deformable ones to cater to the movement of the device as calculated in the finite element module. Furthermore, the energy equation as described in [37,53] is calculated in the finite volume model. The fluid conditions assumed in these analyses were laminar in nature. This is because the scenarios under investigation are predominantly governed by natural convection events dominated by buoyancy forces at the FSI interface. The convection coefficient is calculated within the finite volume domain using Equation (11) in [37]. Two fluids are being assessed in this work, namely air and deionised (DI) water. When considering air in the analysis, the fluid was set as an incompressible ideal gas. In the case of water, temperature-related variables for density and viscosity were used in the analyses; the respective data points were extracted from the literature [54]. The properties of both may be seen in Figure 8 and Table 3.

When invoking ideal gas conditions in Fluent software, the density of the fluid ρ is calculated using Equation (2):

(2) ρ=pOPRMwT

where R is the universal gas constant, $M_{w}$ is the molecular weight of the fluid, $p_{\mathrm{OP}}$ is the operating pressure, and T is the temperature of the fluid [53,55].

Additional boundary conditions that are implemented in the finite volume model include:

- The bottom surface of the domain, which is assumed to be a fixed wall clamped at a constant temperature of 22 °C;
- The five extremities of the domain, which are assumed to be pressure outlets with a gauge pressure of 0 bar. Re-entry of the fluid is allowed here, with a temperature of 22 °C.

#### 4.2.3. Data Transfer Setup

Given that thermo-mechanical coupling is invoked in the proposed methodology, more variables associated with data transfer are possible here when compared with those used in [37]. Table 4 includes a comparison of the data transfer variables together with their source and target modules for thermal and thermo-mechanical coupling.

With reference to Table 4, the proposed method now also caters to mechanical variables. Among them are the displacement of the device at the FSI surfaces as calculated in the finite element module. These displacement data are transferred to the finite volume domain per iteration, and as a result, a force is developed in the finite volume due to the viscous effects of the fluid. This force is then relayed back to the finite element domain together with the thermal-related variables.

One limitation of this proposed method is that no electrical coupling is performed between the finite element and the finite volume domains. Such a function would be a very advantageous for numerical models concerning biomedical applications because the target fluid is typically an aqueous solution with a relatively large electrical conductivity. To the best of the authors’ knowledge, this function is unfortunately not currently supported.

Because the finite element domain for this method is discretised into elements that have electrical, thermal, and structural degrees of freedom, the virtual characterisation of the devices are possible via postprocessing routines that are directly within the finite element environment. Virtual characterisation is possible following successful convergence of all three components in the analysis set, that is, the finite element module, the finite volume module, and the system coupling algorithm.

## 5. Experimental Testing

### 5.1. General

This section describes a series of tests that were performed so as to experimentally characterise the electro-thermo-mechanical performance of the devices in both air and DI water. The numerical model presented is then validated. All experimental testing described here was performed using the MEMS-dedicated testing equipment Cascade Microtech Summit 11 K/12 K B-series station (Cascade Microtech Inc., Beaverton, OR, USA). The station included an in-built microscopy system, which made displacement measurements possible. In all cases, the devices were powered by applying a potential across the anchors of the primary V-shaped drivers. This was possible with the use of x, y, and z micro-positioners that were mounted with 10 µm diameter voltage probes. The probes were brought into contact with the pad metal present at the anchor locations as described in [8]. In all testing scenarios, five measurements were taken per applied volt so as to ensure measurement repeatability. Moreover, the standard deviation was calculated for each set, and this was used to define the measurement error. Note that although there is currently no means at the authors’ disposal to accurately measure out-of-plane deformations, no significant loss of optical focus was observed at the tips during testing, therefore suggesting that out-of-plane deformations are negligible.

### 5.2. Testing in Air

When testing in air, all experiments were performed using a DC source (MCP M10-QS305, MCP Lab Electronics, Shanghai, China). Table 5 illustrates the voltage ranges that were used for the individual devices. All tests were carried out in increments of 0.5 V.

**Figure 9:**
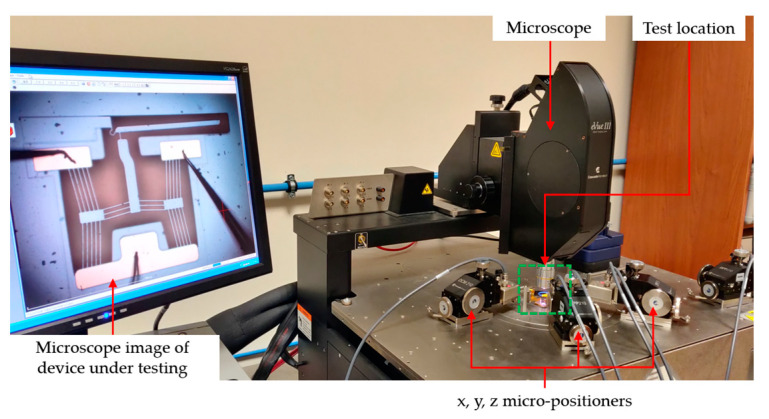
Typical experimental setup for testing in air.

Note that not all devices were tested using equal ranges as the devices that exhibit the largest displacement resulted in out-of-range motions, leading to contact with adjacent SOI material when loaded with values greater than the ones tabulated in Table 5. The reason for this is that the devices in the ‘as manufactured’ state exhibited an unpredicted permanent deformation, i.e., when in the rest (unpowered) condition, which was potentially brought about by the manufacturing process itself. The resulting displacements were optically measured via the station’s inbuilt microscopy system. A typical experimental setup may be seen in Figure 9. Note that for testing the devices in air, no prior sample preparation was performed.

### 5.3. Frequency Independence Study

In order to understand the behaviour of the devices at high-frequency input power, a frequency independence study was performed in air prior to proceeding with testing in DI water. Performing this test is an important task because one may confirm that the device’s mechanical displacement for a given RMS voltage of an AC input signal is similar to when it is actuated using a DC voltage of the same value. The setup for this testing procedure was similar to that as described in Section 5.2 with a few differences. Firstly, the DC power source was replaced by a signal generator (Aim & Thurlby Thandar Instruments, TG550, Huntingdon, UK). Electrical current measurements were excluded from this exercise as the tip displacement being comparable to when the device was activated using a DC source was set as the success criterion. For all devices, a 100 kHz square waveform having a mean voltage of 0 V was applied. Peak voltage values were swept from 0 V to 4 V in increments of 0.5 V. Note that a modal analysis was performed in ANSYS Mechanical™ to identify the device modes and ensure that the applied signal frequency did not excite any resonant modes.

### 5.4. Testing in DI Water

**Figure 10:**
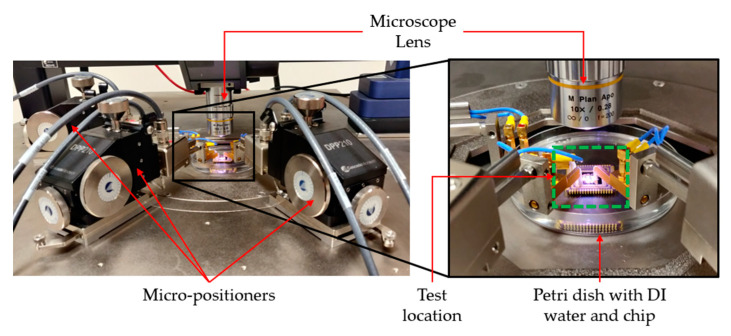
Experimental setup of testing DI water.

The main difference between testing in DI water and in air during the frequency independence study is that polymer Petri dishes were used to contain the liquid. The remaining testing setup and parameters used to experimentally characterise the devices’ function in fully submerged conditions were identical to that explained in Section 5.3. Refer to Figure 10 for a detailed view of a chip submerged in liquid under testing.

Prior to testing, samples of the DI water were extracted, and their electrical conductivity was measured. In all instances, the measured resistivity was found to be 2.4 × 10^9^ µ·Ω·m. Furthermore, for this testing scenario, device preparation was performed as follows:

i. The devices were first subjected to a 10-minute soak in undiluted isopropyl alcohol (IPA);
ii. The devices were then thoroughly rinsed three consecutive times in DI water;
iii. Finally, the devices were submerged again in DI water for testing.

## 6. Results and Discussion

This section is dedicated to presenting the results that were obtained numerically as well as experimentally. Some insight pertaining to the observations made during the analyses shall also be presented. The results shall be correlated with the specifications described in Section 1.1, and the devices’ potential for underwater biomedical applications involving human RBC characterisation is validated.

### 6.1. Electro-Thermal Performance

**Figure 11:**
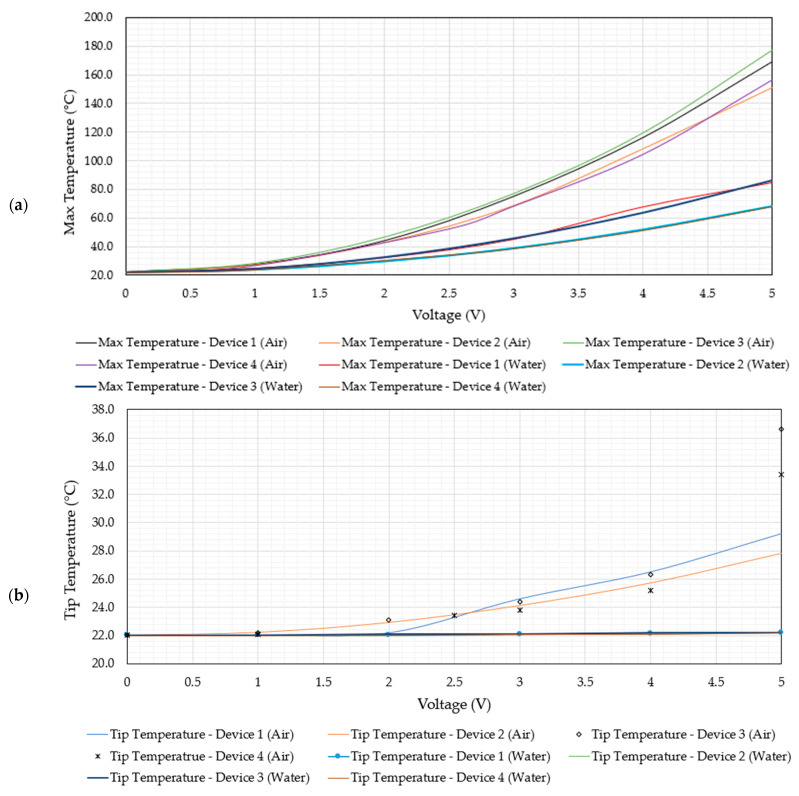
Numerically calculated steady-state temperature versus voltage graphs at (**a**) primary driver maximum temperature region; (**b**) device tip. Note that in the case of water, the voltage is assumed to be RMS.

Recall the thermally-oriented specifications imparted onto MEMS actuators for underwater human RBC characterisation activities, where the tip (test location) shall not exceed a maximum steady-state operating temperature of 40 °C and no location on the device shall exceed the boiling point of water. Within this section, the electro-thermal performance of the MEMS ETAs described above is presented, and the device suitability for these thermal specifications is demonstrated. The thermal performance of the devices was determined purely numerically due to the current unavailability of the necessary equipment at the authors’ disposal. Figure 11 includes plots of the apex and tip temperature versus voltage for all devices in both air and DI water.

With reference to Figure 11, it is evident that all devices fulfil the requirements concerning underwater operation in that up to an input voltage of 5 V, all devices exhibit maximum temperatures below 87 °C and tip temperatures never exceeding 22.2 °C. Table 6 shows the temperature percentage difference between operation in air versus in water at both the tip as well as maximum temperature location of all devices.

**Figure 12:**
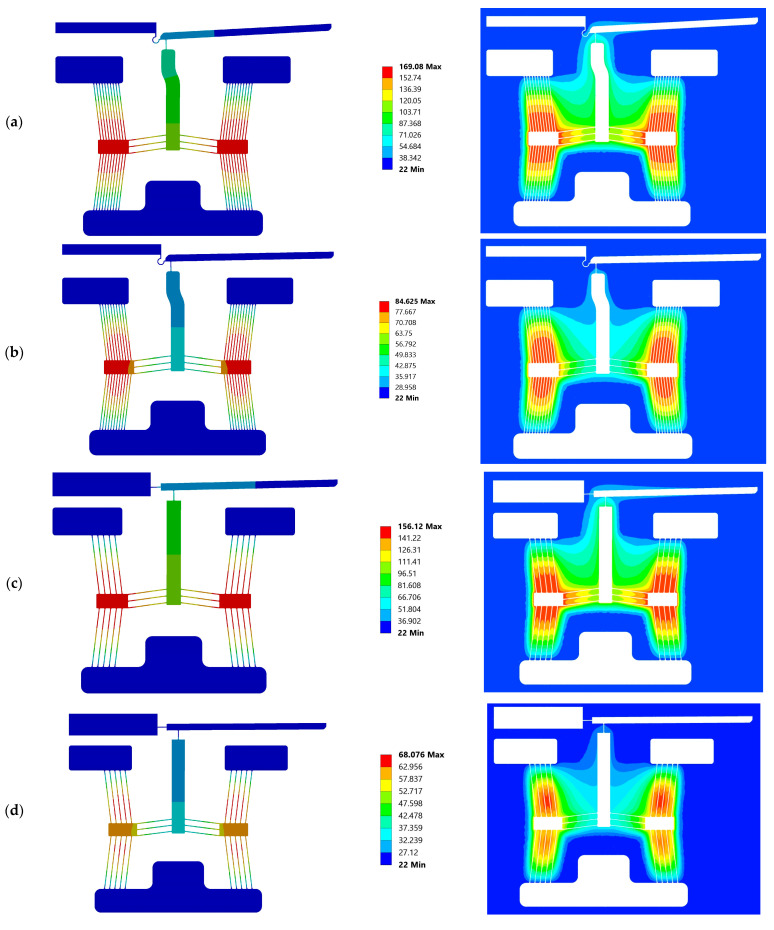
Numerical temperature contours in the finite volume domain (**left**) and corresponding finite volume domain (**right**) of (**a**) device 1 in air, (**b**) device 1 in water, (**c**) device 4 in air, and (**d**) device 4 in water, all at an input voltage of 5 V (or V_RMS_ in the case of water). Legends are temperature in °C.

Devices 1 and 3 are seen to generate the highest maximum temperature when compared with devices 2 and 4 in both media. The reasons for this are twofold: because they are composed of 10 primary beams per side of each individual primary driver, their total electrical resistance is half that of devices 2 and 4, thus allowing for larger Joule heating per supplied volt; and their tighter-spaced primary beams imply more sensible heat, as there is a tendency to lose less heat energy via natural convection. This is also reinforced by the data tabulated in Table 6, whereby devices 1 and 3 experience a lower drop in maximum temperature when operating in water compared with devices 2 and 4. It is also worth noting that the tip temperatures are less affected by the change in medium when compared with the maximum temperature, as exhibited at the primary driver. This is attributed to the fact that the devices incorporate a large thermal path between their apexes and the tip so as to fulfil the thermal requirement highlighted in Section 1.1.2. This large thermal path means that the tip at the steady state shall attain much lower temperatures per applied volt than the driver and is therefore less prone to heat losses by convection. Figure 12 includes temperature plots in the finite element and corresponding finite volume domains of devices 1 and 4 in both air and water. Unless otherwise specified, the discussion here (within the current paragraph) concerning device 1 and 4 also applies to devices 3 and 2, respectively. With reference to Figure 12, the enhanced ability of device 1 to confine higher temperatures at the primary beams when compared with device 4 is evident. In the case of device 4, the temperature gradients across primary beam pairs on the horizontal are clearly evident in air (Figure 12c), whereas this is not the case when considering device 1 (Figure 12a). Considering device 1 in water on the other hand, it may be seen that the outer primary beams maintain lower temperatures than the inner beams; this may be pinned down to the fact that because the substrate was assumed to maintain a constant temperature of 22 °C, the water is clamping the beam temperature to the mentioned substrate, which is acting as a heat sink. In fact, in all device instances, the bottom primary beam set is observed to have lower temperatures as they are designed closer to the substrate material (recall Figure 4). Device 4 exhibits a more pronounced heat loss in water when compared with air as rather than simply losing a good amount of heat to the substrate, the wider-spaced beams allow for more heat loss via natural convection, so much so that the full potential of the primary V-shaped mechanism is not achieved at an input voltage of 5 V. With reference to Figure 12d, it is observed that the primary apexes of device 4 do not reach the maximum temperature at steady-state operation as convection between the primary beams is dominating over conduction through the semiconductor.

### 6.2. Structural Performance

The device structural performance was characterised by monitoring the tip displacements with the input voltage in both air and DI water. The numerically and experimentally obtained values for the tip displacement are compared. The success criterion here is the ability to achieve tip displacements of at least 7 µm in fully submerged conditions. This was set because of the device/devices’ criteria/specifications outlined in Section 1.1.1, where a human RBC may be compressed by as much as 7 µm.

#### 6.2.1. Device Structural Performance in Air

**Figure 13:**
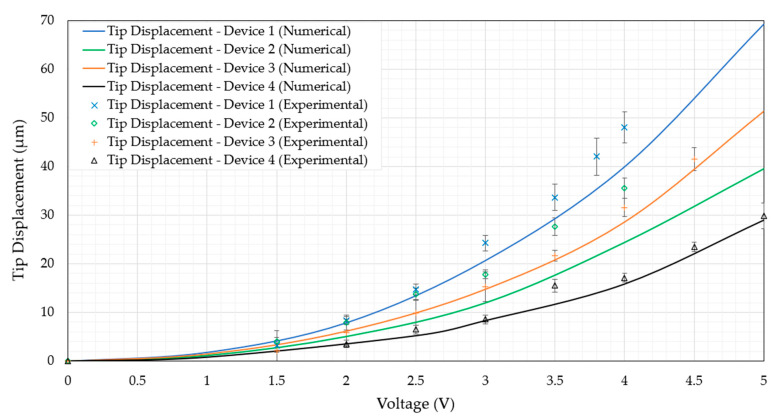
Experimental and numerical tip displacement vs. voltage graph of all devices in air and at steady-state operation when subjected to an external DC source. Note that the experimental tip displacements of devices 1 to 3 do not proceed up to 5 V as a clash condition with the surrounding SOI was present at input voltages that were higher than those presented.

Within this section, the device performance in air when subjected to an external DC source is evaluated. Although not directly related to the function of primary focus in this work (that is, the device function when submerged in aqueous conditions), testing in air is an imperative task for two main reasons. Firstly, the devices may be used for functions requiring mechanical compression of micro-objects other than biological cells, which may be performed in air. Secondly, the results from this section shall serve a baseline to the upcoming frequency independence study, where the device’s function when subjected to a high-frequency AC source shall be compared with its function when subjected to a DC source. The numerically derived as well as experimentally measured tip displacement versus voltage graphs of all devices in air are shown in Figure 13.

With reference to Figure 13, it is evident that the numerical models are in good agreement with the experimental measurements. The numerical models underpredict the tip displacement, and the reason for this may be that the ambient temperature may have increased from the desired 22 °C. Furthermore, there may be dimensional variations that are brought about by process-related tolerances, which were not taken into account in the numerical models where the device geometry at the mean conditions was considered. This mild underprediction may be seen as a beneficial error as it serves as a safety margin during the design and development stages. The thermo-mechanical FSI numerical model has therefore proved to be a suitable candidate for device function in air. It is also worth noting that devices 1 and 3 generate the largest tip displacements per volt when compared with devices 2 and 4. This can be attributed to the larger maximum temperatures developed in devices 1 and 3, the reasons of which were described in Section 6.1. Moreover, because devices 1 and 3 have twice the amount of beams per side of the primary drivers, they have a higher stiffness to overcome the mechanical resistance that is imparted by the passive secondary amplification mechanism and amplification beam hinges. Then again, the higher displacements exhibited by devices 1 and 2 when compared with devices 3 and 4 is solely due to the fact that devices 1 and 2 have a less stiff amplification beam hinge structure, which offers less mechanical resistance and therefore allows for larger tip displacement.

#### 6.2.2. Frequency Independence Study in Air

**Figure 14:**
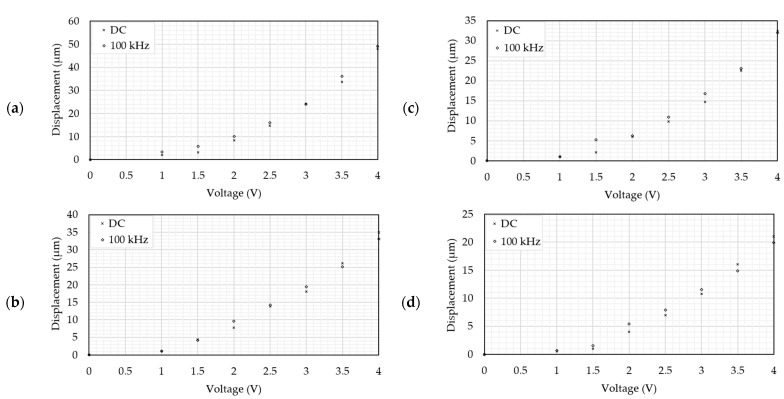
Tip displacement versus voltage graphs of (**a**) device 1, (**b**) device 2, (**c**) device 3, and (**d**) device 4. In the case of the high frequency analysis (100 kHz), the voltage axis is RMS.

The frequency independence study bridges the gap between the device operation in air and in water. This activity was performed purely experimentally and in air. In all instances and with reference to Section 1.1.3, a 100 kHz square waveform with a mean voltage of 0 V was used to power the devices. The tip displacement with an RMS voltage was then compared with the tip displacement versus an equivalent DV voltage. Figure 14 includes plots of tip displacement against voltage for all devices.

The data presented in Figure 14 prove that the devices’ response to an RMS input is practically identical to that supplied by a DC source. This provides confidence that such a source is suitable from a device operation perspective and may be used for testing in DI water.

#### 6.2.3. Device Structural Performance in DI Water

**Figure 15:**
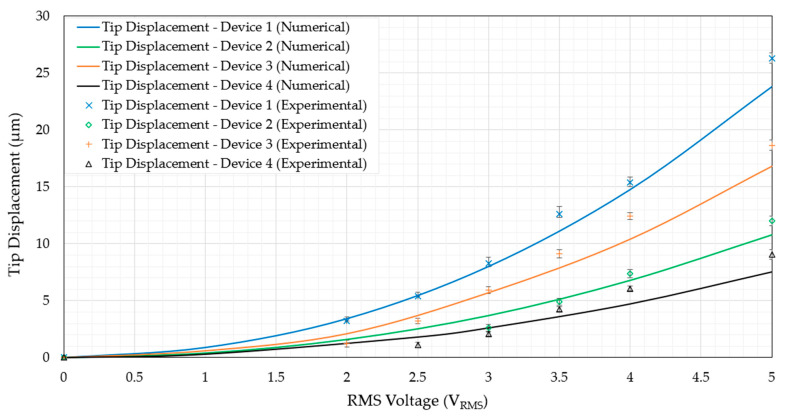
Experimental and numerical tip displacement versus RMS voltage of all four devices.

This subsection is dedicated to demonstrating the devices’ mechanical performance in fully submerged conditions. Similarly to the performance in air, the tip displacement was the key performance parameter, and the success criterion was the ability to displace by at least 7 µm without exceeding the temperature values discussed in Section 1.1.2 and Section 1.1.3. Tip displacement measurement in DI water was performed experimentally and calculated numerically. Figure 15 includes the tip displacement versus the voltage graphs of all devices in DI water.

Similarly to the trend and behaviour seen in the case of device function in air, the numerical model is in good agreement with the experimental findings, again being slightly underpredictive. The magnitude of underprediction here is somewhat less than that seen in operation in air, and this is attributed to the fact that the specific heat capacity C_p_ of water is approximately four times higher than that of air, therefore making water less susceptible to temperature fluctuations with ambient air. Moreover, devices 1 and 3 can again be seen to generate the largest tip displacements for the same reasons as elaborated in Section 6.2.1 in that their primary mechanisms are composed of twice the amount of primary beams working in parallel. This makes their ability to overcome the mechanical resistance of the remaining structure better. Additionally, their total electrical resistance is less than that of devices 2 and 4, and they lose less heat via convection due to their closely spaced primary beams.

With reference to Figure 11 and Figure 15, it has been proven that all four devices presented in this work are suitable candidates for human RBC characterisation in submerged conditions due to the following reasons:

- All four devices operate at maximum temperatures well below 100 °C at an input voltage V_RMS_ of 5 V;
- At V_RMS_ = 5 V, all four devices maintain tip temperatures that are practically identical to the surrounding ambient temperature of 22 °C;
- The tip displacements at 5 V are all above 7 µm. The lowest was device 4, displaying experimental tip displacements of 9.1 µm, while the highest was showcased by device 1 at 26.3 µm.

The tip displacement in DI water as a percentage of that in air was calculated and can be seen in Table 7. Note that the voltage ranges were initiated from 2 V as no measurable tip displacement was generated (experimentally and in DI water) at values below 2 V in the case of all devices. The cells with ‘N/A’ as inputs also indicate points where no measurable tip displacements were obtained.

With reference to Table 7, note that the device’s mechanical function (in terms of tip displacement) in water compared with that in air is significantly higher than the estimated 6 % documented in the literature [31] at all voltage values. Another interesting observation worth noting here is that devices 1 and 3 display higher operational percentages than their counterparts. The reason for this is attributed to their tighter-spaced primary driver beams that contain more heat energy in their vicinity.

### 6.3. Gas Evolution Observation

**Figure 16:**
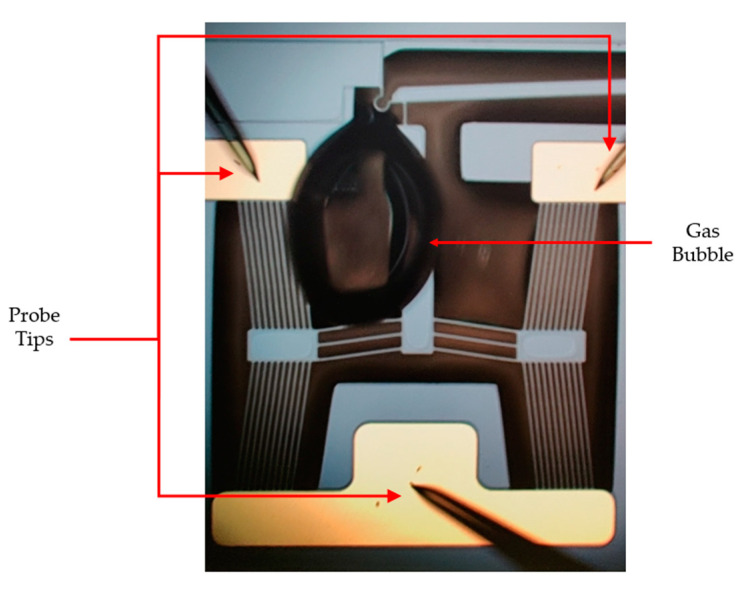
Gas evolution as observed during the experimental testing of the devices in DI water without prior sample preparation. Although the same issue was observed when testing all devices, the device in the image is device 1.

During the initial phases of underwater testing, no sample preparation was being performed. Although a high-frequency input source was being utilised to power the devices, regenerative gas evolution was being observed in all device iterations at voltages as low as 1 V_RMS_. With reference to Figure 16, the bubble formation site was not analogous to what would be expected from electrolysis, as the gas bubble would form somewhere other than the electrode locations. This phenomenon was overcome by rinsing the devices in IPA prior to testing, and this action was introduced as an initial sample preparation task for testing in DI water procedures, as outlined in Section 6.2.3. Unfortunately, no specialised equipment is available at the authors’ disposal to determine the exact constituents of the gas being evolved.

## 7. Conclusions

Throughout this work, four MEMS ETA designs were presented. They were fabricated using the commercially available SOIMUMPs micromachining process and all make use of a cascaded V-shaped driver to impart the necessary displacement when subjected to an external power source. Devices 1 and 3 both had primary drivers that were composed of 10 equally spaced beams on either side of the primary apex, whereas devices 2 and 4 have primary drivers with 5 beams. Devices 1 and 2 have identical amplification mechanisms, which differs from that of devices 3 and 4 [8].

In contrast to the work presented in [37] where thermal coupling was calculated, this work presented a numerical modelling methodology invoking thermo-mechanical, fluid–structure interaction coupling, which is capable of accurately modelling MEMS ETA function in various media such as air and water. This methodology was the main modelling method adopted during the design and development of the four devices presented in this research paper. Despite calculating for electrothermal phenomena in the finite element domain, one major drawback of the proposed numerical modelling technique is that electrical coupling between the finite element and finite volume domains is currently not possible to the best of the authors’ knowledge.

The critical variables of primary focus during the post-processing routines were thermal and structural in nature. The thermally related specifications included a net maximum temperature (on the devices’ entire structure) not exceeding 100 °C and a tip temperature not exceeding 40 °C when submerged in water. The numerical models shed light on the fact that all devices adhered to this set of specifications up to an input voltage of 5 V_RMS_, where devices 1 and 3 exhibited the highest maximum temperatures of 84.6 °C and 86.2 °C, respectively; meanwhile, devices 2 and 4 both exhibited maximum temperatures of 68.1 °C as calculated numerically. The higher maximum temperature generated by devices 1 and 3 was deduced to be primarily due to their lower electrical resistance and their tighter-spaced primary beams, which allow for weaker convection events. The tip temperatures of all devices in water were calculated to be as low as 22.2 °C at an input voltage of 5 V_RMS_, and this is due to the relatively large thermal path inbuilt into all devices, making passive cooling via natural convection in water sufficient for the testing and manipulation exercises of human RBCs. Recall that the structural specification set was a minimum tip displacement of 7 µm so as to impart the necessary deformation along the major axis of a human RBC. The numerically calculated tip displacement of devices 1 through 4 were 23.8 µm, 10.8 µm, 16.8 µm, and 7.5 µm, respectively, at an input voltage of 5 V_RMS_. Devices 1 and 3 generated the highest displacements because their primary drivers are composed of 10 beams per side working in parallel, therefore making them more capable of overcoming the mechanical resistance from the passive features composing the rest of the device. Furthermore, their tighter spaced primary beams contain more heat in their vicinity, thus allowing them to generate the higher temperatures already discussed. This in turn allows for larger thermal expansion and hence total tip displacement. The main difference in tip displacement between devices 1 and 3 is solely attributed to their difference amplification beam hinge features, where device 3 has a stiffer hinge configuration when compared with device 1. This final point also holds for the difference in the tip displacement calculated between devices 2 and 4, as device 4 shares a common hinge configuration with device 3 but is otherwise identical to device 2.

Whereas the previous work of [37] only concerned itself with numerical modelling, all devices presented here were structurally and mechanically tested experimentally using MEMS-dedicated testing equipment. This testing sequence also validated the numerical modelling approach in that the numerical and experimental device tip displacements were found to be in good agreement with one another in both air and DI water. In both media, the numerical models were found to underpredict the tip displacements. The magnitude of underprediction in air was determined to be larger than that of water. A possible reason for this is that ambient air temperature fluctuations are more pronounced in the devices’ tip displacement when compared with testing in water, and this may be explained by the larger specific heat capacity of water. A frequency independence study was performed in air, whereby all devices’ tip displacements when excited by an external DC source were compared with the tip displacements that were generated when the devices were powered by an equivalent 100 kHz square wave input with a mean voltage of 0 V. It was determined that the difference in device response to the RMS input of the higher frequency input was negligible when compared with that of the DC input. This instilled confidence in testing using such a high-frequency input prior to testing in water, whereby a DC source of over 1.23 V is not suitable due to the electrochemical phenomena such as electrolysis of water. The tip displacement in water as a percentage of that in air was calculated, and devices 1 and 3 showed the highest percentage compared with devices 2 and 4 due to their ability to maintain higher primary beam temperatures. Moreover, it was deduced experimentally and numerically that all devices are suitable for the intended function of performing mechanical characterisation of human RBCs, as devices 1 to 4 generated tip displacements of 26.3 µm, 12.02 µm, 18.65 µm, and 9.06 µm, respectively, at an actuation voltage of 5 V_RMS_ in water. The experimentally measured tip displacement values were observed to be higher than those calculated numerically. This was primarily attributed to either the fluid temperature not coinciding exactly with that of the numerical models as well as the geometrical tolerances in the physical devices, which were also not considered in the numerical models.

A miscellaneous observation attributed to device operation in DI water was also briefly highlighted, whereby gas evolution was noticed even with a high-frequency operation of the devices. The IPA rinse prior to submersion completely eliminated the gas evolution.

## Figures and Tables

**Table 1:** Material properties of the SOI and pad metal layers. Values were taken from [35] aside from the electrical resistivity of the SOI, which was extracted from [8]. The designations ‘x’ and ‘y’ refer to the in-plane, whereas ‘z’ refers to the out-of-plane. All properties are assumed to be at a reference temperature of 22 °C. © 2023 IEEE. Reprinted with permission from T. Sciberras, P. Mollicone, M. Demicoli, I. Grech, N. Sammut, and B. Mallia, “Experimental and Numerical Analysis of MEMS Electrothermal Actuators with Cascaded V-shaped Mechanisms,” *2022 Symposium on Design*, *Test*, *Integration and Packaging of MEMS/MOEMS (DTIP)*, Pont-a-Mousson, France, 2022, pp. 1–5 [8].

Property Designation	SOI	Pad Metal
Shear modulus, G [GPa]	G_yz_ = G_zx_ = 79.6, G_xy_ = 50.9	N/A
Young’s modulus, E [GPa]	E_x_ = E_y_ = 169, E_z_ = 130	57
Poisson’s ratio, ν	ν_yz_ = 0.36, ν_zx_ = 0.29, ν_xy_ = 0.064	0.35
Thermal conductivity, k [W/m·K]	148	297
Coefficient of thermal expansion, α [µm/m·K]	Refer to Figure 2	N/A
Density [g/(cm)^3^]	2.50	19.30
Specific heat capacity, c [J/kg·K]	712	128.7
Electrical resistivity, ρ [µ·Ω·m]	125	2.86 × 10^−2^

**Table 2:** Geometric parameters of MEMS ETA devices as extracted from [8]. © 2023 IEEE. Reprinted with permission from T. Sciberras, P. Mollicone, M. Demicoli, I. Grech, N. Sammut, and B. Mallia, “Experimental and Numerical Analysis of MEMS Electrothermal Actuators with Cascaded V-shaped Mechanisms,” *2022 Symposium on Design*, *Test*, *Integration and Packaging of MEMS/MOEMS (DTIP)*, Pont-a-Mousson, France, 2022, pp. 1–5 [8].

Variable	Value			
	Device 1	Device 2	Device 3	Device 4
Number of beams per side (primary V-shaped mechanism)	10	5	10	5
Number of beams per side (secondary V-shaped mechanism)	3	3	3	3
Primary beam width, w_B1_ (µm)	6	6	6	6
Primary beam spacing, w_G1_ (µm)	14	34	14	34
Secondary beam width, w_B2_ (µm)	6.6	6.6	6.6	6.6
Secondary beam spacing, w_G2_ (µm)	40	40	40	40
Distance between anchors of primary V-shaped mechanism, L_1_ (µm)	920	920	920	920
Distance between primary apexes (at rest and 22 °C), L_2_ (µm)	640	640	640	640
Pre-bend angle, θ (°)	7	7	7	7

**Table 3:** Material properties of fluids as used in the finite volume domain. Properties as extracted from [54]. Unless otherwise specified, properties are assumed to be at a temperature of 22 °C.

Property	Fluid	
	Air	Water
Density	Refer to Equation (2)	Refer to Figure 8
Specific heat at constant pressure, J/kg·K	1006.43	
Thermal Conductivity, W/(m·K)	0.02602	
Viscosity, kg/(m·s)	1.7894 × 10^−4^	10.03 × 10^−4^
Molecular weight, kg/kmol	28.966	N/A

**Table 4:** Data transfers as set up in both the thermal and thermo-mechanical coupling simulations, as adapted from [37].

Data Source	Target Module	Source Variable	Affected Target Variable	Thermal Coupling [37]	Thermo- Mechanical Coupling
Finite Volume	Finite Element	Heat Transfer Coefficient	Convection Coefficient	✓	✓
Finite Volume	Finite Element	Near Wall Temperature	Convection Reference Temperature	✓	✓
Finite Element	Finite Volume	Temperature	Temperature	✓	✓
Finite Volume	Finite Element	Force	Force	N/A	✓
Finite Element	Finite Volume	Incremental Displacement	Displacement	N/A	✓

**Table 5:** The voltage ranges that were experimentally tested.

Device/s	Voltage Range
1 and 2	0 V–4 V
3	0 V–4.5 V
4	0 V–5 V

**Table 6:** Temperature percentage difference of air against water.

Voltage (V)	Percentage Difference (%)							
	Tip				Maximum			
	Device 1	Device 2	Device 3	Device 4	Device 1	Device 2	Device 3	Device 4
0	0	0	0	0	0	0	0	0
1	0.14	0.9	0.9	0.45	10.40	12.18	13.33	12.18
2	0.90	4.01	4.42	/	31.36	35.68	34.94	/
2.5	/	/	/	5.94	/	/	/	41.94
3	10.84	8.66	9.89	7.41	50.23	55.22	50.90	55.33
4	17.88	15.06	16.91	13.11	52.72	70.42	60.98	67.95
5	27.24	27.8	48.98	40.29	66.61	75.62	69.20	78.51

**Table 7:** Experimental tip displacement in DI water as a percentage of experimental tip displacement in air.

Voltage	Percentage (%)			
	Device 1	Device 2	Device 3	Device 4
2	38.50	N/A	20.76	N/A
2.5	36.54	N/A	32.42	17.00
3	34.12	14.63	38.69	24.24
3.5	37.44	17.62	42.00	27.52
4.0	32.00	20.69	39.30	35.53

## Data Availability

Data sharing not applicable.

## Abbreviations

The following abbreviations are used in this manuscript:

BC	Boundary condition
ETA	Electrothermal actuator
FSI	Fluid–structure interaction
MEMS	Microelectromechanical system
SOI	Silicon-on-insulator
RBC	Red blood cell
DI	Deionised
